# Association of QRS Notch Resolution During LV‐Only Fusion Pacing With Early Left Ventricular Reverse Remodeling After Cardiac Resynchronization Therapy

**DOI:** 10.1002/joa3.70443

**Published:** 2026-08-09

**Authors:** Junji Morita, Yuhei Kasai, Yumetsugu Munakata, Takayuki Kitai, Kei Murakami, Kazunari Mitsuishi, Daisuke Hachinohe, Yusuke Kondo

**Affiliations:** ^1^ Department of Cardiology Sapporo Cardiovascular Clinic Sapporo Hokkaido Japan; ^2^ Department of Clinical Technology Sapporo Cardiovascular Clinic Sapporo Hokkaido Japan; ^3^ Department of Cardiovascular Medicine Chiba University Graduate School of Medicine Chiba Japan

**Keywords:** cardiac resynchronization therapy, left bundle branch block, left ventricular reverse remodeling, left ventricular‐only pacing, QRS notch resolution

## Abstract

**Background:**

Cardiac resynchronization therapy (CRT) improves outcomes in patients with heart failure and left bundle branch block (LBBB); however, many patients remain non‐responders. Identifying effective electrical resynchronization early remains an important clinical need.

**Objective:**

To investigate the association between resolution of V5–V6 R‐wave notching/slurring during left ventricular‐only pacing (LV‐only pacing), combined with an LV‐only pacing percentage ≥ 50% at 1 month, and early left ventricular reverse remodeling in patients with complete LBBB (CLBBB) undergoing CRT.

**Methods:**

We retrospectively studied 49 patients with CLBBB who underwent CRT. Patients with resolution of V5–V6 R‐wave notching/slurring during LV‐only pacing at implantation and an LV‐only pacing percentage ≥ 50% at 1 month were classified as the R‐Notch Resolution group; all others were classified as the Non–R‐Notch Resolution group. The primary endpoint was left ventricular reverse remodeling at 1 month, defined as a ≥ 15% reduction in left ventricular end‐systolic volume (LVESV) from baseline. Logistic regression analysis was performed to identify associated factors.

**Results:**

Fifteen patients were classified into the R‐Notch Resolution group and 34 into the Non–R‐Notch Resolution group. One‐month left ventricular reverse remodeling occurred more frequently in the R‐Notch Resolution group (80.0% vs. 47.1%). In multivariable logistic regression analysis, R‐Notch Resolution group status remained independently associated with 1‐month left ventricular reverse remodeling.

**Conclusion:**

Resolution of V5–V6 R‐wave notching/slurring during LV‐only pacing, combined with an LV‐only pacing percentage ≥ 50% at 1 month, was independently associated with early left ventricular reverse remodeling after CRT.

## Graphical abstract

QRS notch/slur resolution during LV‐only fusion pacing, combined with an LV‐only pacing percentage ≥ 50% at 1 month, was independently associated with early left ventricular reverse remodeling and may help guide post‐implant CRT optimization.

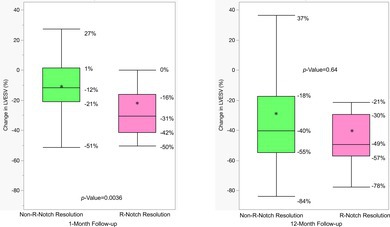

## 1 Introduction

Cardiac resynchronization therapy (CRT) is an established treatment for patients with heart failure and left bundle branch block (LBBB) [1, 2, 3]. Nevertheless, approximately 30%–40% of patients remain non‐responders [4, 5, 6, 7], highlighting the need for better tools to identify patients likely to benefit from CRT and to guide device programming. Left ventricular reverse remodeling (LVRR) is closely associated with long‐term outcomes [8].

Several electrocardiographic (ECG) markers have been proposed as predictors of CRT response, including post‐implant QRS narrowing and simplification of QRS morphology [9]. Acute shortening of QRS duration immediately after CRT implantation has been associated with LVRR and improved clinical outcomes [10]. A characteristic ECG feature of complete LBBB is mid‐QRS notching/slurring in the lateral leads (I, aVL, V5–V6). This morphology is incorporated in strict LBBB definitions and has been associated with greater CRT response, consistent with the concept of ‘true/complete LBBB’ [11, 12, 13, 14].

Recently, left ventricular‐only pacing (LV‐only pacing), a form of fusion pacing that aims to achieve more physiological resynchronization, has attracted increasing attention. Studies have demonstrated non‐inferior improvements in cardiac function compared with conventional biventricular pacing (BVP), particularly when a high rate of LV‐only fusion pacing is maintained [15, 16, 17]. However, the relationship between changes in QRS notching or slurring under LV‐only pacing and early reverse remodeling after CRT has not been clarified, because most previous studies have evaluated response at 3–6 or 12 months [4, 8, 18, 19]. Therefore, we investigated CRT recipients with complete LBBB (CLBBB) to assess whether patients in whom R‐wave notching/slurring in V5–V6 had resolved under LV‐only pacing at implantation and who achieved an LV‐only pacing rate ≥ 50% at 1 month (R‐Notch Resolution group) were more likely to show 1‐month LV reverse remodeling after CRT implantation.

## 2 Methods

### 2.1 Study Design and Population

This was a single‐center retrospective observational cohort study. We screened consecutive adult patients who underwent CRT device implantation at our institution between 2021 and 2024 and met the following criteria. Inclusion criteria were: age ≥ 18 years; chronic heart failure on guideline‐directed medical therapy; left ventricular ejection fraction (LVEF) ≤ 35%; New York Heart Association (NYHA) functional class II or higher; sinus rhythm with complete left bundle branch block (CLBBB); CLBBB was defined, in accordance with current guidelines and previous reports [1], as a QRS duration ≥ 120 ms with R‐wave notching or slurring present in at least two of leads I, aVL, and V5–V6. Exclusion criteria were: upgrade procedures from a pre‐existing pacemaker or implantable cardioverter‐defibrillator (ICD); CRT implantation primarily for high‐grade atrioventricular block with reduced LVEF; CRT in patients without LBBB; absence of follow‐up transthoracic echocardiography and device interrogation data at 1 month after implantation.

The study protocol was approved by the institutional ethics committee and the study was conducted in accordance with the Declaration of Helsinki. Because of the retrospective design, informed consent was obtained using an opt‐out process via the institutional website and related public information materials.

### 2.2 Device Implantation and Programming

All procedures were performed via a prepectoral approach under local anesthesia and moderate sedation with propofol. The right atrial lead was positioned in the right atrial appendage, the right ventricular lead in the mid‐septal or apical region of the right ventricle, and the left ventricular lead was advanced via the coronary sinus and placed in a lateral or posterolateral vein targeting the latest activated region. Devices were manufactured by Medtronic (Minneapolis, MN, USA), Biotronik (Berlin, Germany), or Abbott (Abbott Park, IL, USA), and the majority were CRT‐defibrillators (CRT‐D). Post‐implant CRT programming was performed during the implantation hospitalization as part of routine clinical practice and was not based on a prespecified study protocol. In Medtronic devices, the AdaptiveCRT “BiV + LV” setting was selected, allowing automatic adjustment between LV‐only pacing and biventricular pacing according to intrinsic atrioventricular conduction and device algorithm behavior. In general, LV‐only pacing is favored when intrinsic AV conduction is sufficiently preserved and stable, allowing fusion between native right ventricular activation and LV‐paced activation, whereas biventricular pacing is selected when intrinsic AV conduction is prolonged, unstable, or unsuitable for effective fusion. In Biotronik devices, AutoAdapt was activated, allowing automatic adaptation between LV‐only pacing and biventricular pacing according to intrinsic conduction and device algorithm behavior. In patients with Abbott devices, the decision to use LV‐only pacing was made at the discretion of the treating physician. SyncAV was used for AV delay optimization with the aim of achieving QRS narrowing rather than as an LV‐only pacing algorithm. During follow‐up, programming was adjusted as needed based on 12‐lead ECG findings, clinical symptoms, echocardiography, and device interrogation. Programming was not prospectively directed toward achieving QRS notch/slur resolution. Programming characteristics and vendor‐specific optimization algorithms have been described previously [20, 21, 22].

### 2.3 Follow‐Up and Data Collection

Follow‐up evaluations were scheduled at baseline (pre‐implant) and at 1, 3, 6, and 12 months after implantation. At each visit, a 12‐lead electrocardiogram, transthoracic echocardiography, and device interrogation (remote or in‐clinic) were performed. In patients with paroxysmal atrial fibrillation, only ECG and echocardiographic data obtained during sinus rhythm at the scheduled time points were used for analysis.

### 2.4 ECG Assessment

For each patient, 12‐lead ECGs recorded at baseline, immediately after implantation, and at 1 month were reviewed. The presence of R‐wave notching or slurring in leads V5–V6 was visually assessed by two board‐certified cardiologists independently, and discrepancies were resolved by consensus.

### 2.5 Echocardiographic Assessment

Transthoracic echocardiography was performed using the same ultrasound system and standardized protocol. Left ventricular volumes and ejection fraction were calculated using the biplane Simpson method. Left ventricular reverse remodeling (CRT response) was defined as a ≥ 15% reduction in left ventricular end‐systolic volume (LVESV) from baseline and/or a ≥ 5% increase in LVEF. In the primary analysis, we focused on changes in LVESV and calculated the relative percentage change in LVESV at 1, 3, 6, and 12 months after implantation.

### 2.6 Device Data

From device diagnostics, we obtained the LV‐only pacing percentage and the total ventricular pacing percentage at 1 month after implantation. LV‐only pacing percentage was defined as the percentage of total device‐recorded time during which LV‐only pacing was delivered. Thus, an LV‐only pacing percentage < 50% did not indicate insufficient overall CRT pacing; rather, it indicated that LV‐only pacing accounted for < 50% of the total device‐recorded time, with the remaining time consisting of BiV pacing and/or intrinsic ventricular conduction, depending on device programming and intrinsic atrioventricular conduction.

### 2.7 Group Classification

Patients were classified into two groups based on the 12‐lead ECG recorded at implantation under LV‐only pacing and the device data obtained at 1 month after implantation.

#### 2.7.1 R‐Notch Resolution Group (Resolved Notch or Slur With LV‐Only Pacing Group)

**FIGURE 1:**
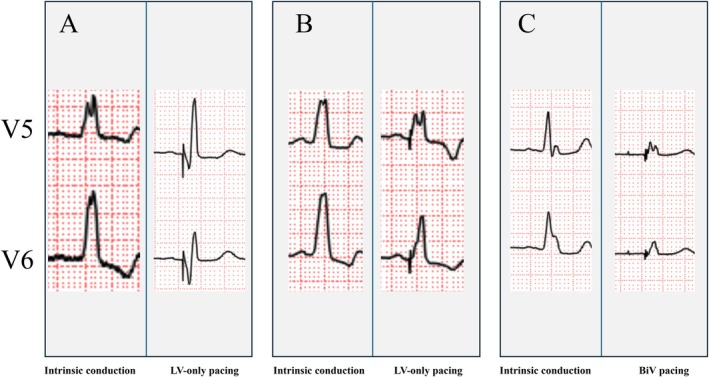
Representative electrocardiographic changes in the R‐wave notch during cardiac resynchronization therapy. Leads V5 and V6 are shown. In each panel, the left tracing shows intrinsic conduction with baseline QRS notching or slurring, whereas the right tracing shows the corresponding paced QRS morphology. (A) Resolution of the R‐wave notch during left ventricular (LV)‐only pacing. (B) Persistence of the R‐wave notch during LV‐only pacing. (C) Persistence of the R‐wave notch during biventricular (BiV) pacing.

Patients who fulfilled both of the following criteria: R‐wave notching or slurring in leads V5–V6, present at baseline, had resolved on the 12‐lead ECG recorded at implantation under LV‐only pacing (Figure 1); and LV‐only pacing percentage at 1 month was ≥ 50%.

#### 2.7.2 Non–R‐Notch Resolution Group

All remaining patients who did not meet the above criteria were assigned to the Non–R‐Notch Resolution group. Patients in whom the LV‐only pacing function was not activated were, by definition, included in the Non–R‐Notch Resolution group.

### 2.8 Statistical Analysis

Continuous variables were expressed as mean ± standard deviation or median [interquartile range], and categorical variables as counts and percentages. For between‐group comparisons, unpaired *t*‐test were used for continuous variables with approximately normal distributions, and the Mann–Whitney *U* test was applied when normality was not assumed. Categorical variables were compared using the *χ* ^2^ test or Fisher's exact test, as appropriate.

Logistic regression analyses were performed with the presence or absence of left ventricular end‐systolic volume (LVESV) reverse remodeling at 1 month as the dependent variable. Explanatory variables included R‐Notch Resolution group (vs. Non–R‐Notch Resolution group), age, sex, presence of ischemic cardiomyopathy, baseline QRS duration, baseline LVESV, and other clinically relevant covariates. Odds ratios (ORs) and 95% confidence intervals (CIs) were calculated, and a *p* value < 0.05 was considered statistically significant.

## 3 Results

### 3.1 Patient Flow and Group Allocation

**FIGURE 2:**
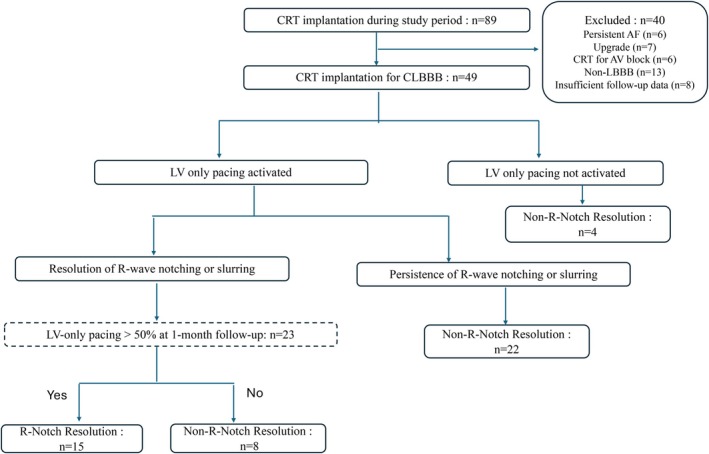
Patient flow and group classification. Flow diagram of patient selection from 89 consecutive CRT implantations during the study period and classification of 49 patients with complete left bundle branch block (CLBBB) into the R‐Notch Resolution (*n* = 15) and Non–R‐Notch Resolution (*n* = 34) groups. Patients with resolution of V5–V6 R‐wave notching/slurring under LV‐only pacing at implantation and an LV‐only pacing percentage ≥ 50% at 1 month were assigned to the R‐Notch Resolution group; all others were classified as the Non–R‐Notch Resolution group.

During the study period, 89 patients underwent CRT implantation at our institution. After excluding patients with persistent atrial fibrillation (*n* = 6), upgrade procedures (*n* = 7), CRT implantation for atrioventricular block (*n* = 6), non‐LBBB QRS morphology (*n* = 13), and those with insufficient follow‐up data (*n* = 8), 49 patients with CLBBB were included in the final analysis (Figure 2). Baseline characteristics of included and excluded patients were generally similar (Table S1). Among these 49 patients, the LV‐only pacing function was not activated in four patients; therefore these were assigned to the Non–R‐Notch Resolution group. Among the remaining 45 patients in whom LV‐only pacing was used, R‐wave notching or slurring in leads V5–V6 resolved in 23 patients. Of these, 15 patients had an LV‐only pacing percentage ≥ 50% and were classified as the R‐Notch Resolution group, whereas the remaining eight were assigned to the Non–R‐Notch Resolution group. The other 22 patients in whom notching/slurring persisted, together with the four patients without LV‐only pacing, comprised the Non–R‐Notch Resolution group, resulting in 15 patients in the R‐Notch Resolution group and 34 in the Non–R‐Notch Resolution group.

### 3.2 Baseline Characteristics

The mean age of the 49 patients was 73.7 ± 8.6 years, and 40 patients (81.6%) were male. The mean LVEF was 29.2% ± 3.6%, median LVESV was 137 [120–169] mL, and mean QRS duration was 153.5 ± 14.9 ms. The underlying etiology of heart failure was non‐ischaemic cardiomyopathy in 34 patients (69.4%) and ischaemic cardiomyopathy in 15 (30.6%). Regarding comorbidities, hypertension was present in 29 patients (59.2%), diabetes mellitus in 12 (24.5%), dyslipidaemia in 32 (65.3%), chronic kidney disease in 32 (65.3%), and paroxysmal atrial fibrillation in 17 (34.7%). Most patients received CRT‐D devices (44 patients, 89.8%), while five patients (10.2%) received CRT‐P. Angiotensin‐converting enzyme inhibitors/angiotensin receptor blockers/ARNI, mineralocorticoid receptor antagonists, and β‐blockers were prescribed in approximately 70%–90% of patients. When baseline characteristics were compared between the R‐Notch Resolution (*n* = 15) and Non–R‐Notch Resolution (*n* = 34) groups, there were no significant differences in age, sex, underlying etiology, comorbidities, or medical therapy. Baseline LVEF, LVESV, and QRS duration were also similar between the two groups. Overall, baseline characteristics were generally similar between the R‐Notch Resolution (*n* = 15) and Non–R‐Notch Resolution (*n* = 34) groups, with no marked differences in demographics, underlying etiology, comorbidities, or medical therapy (Table 1).

**TABLE 1:** Baseline patient characteristics.

	All patients (*n* = 49)	R‐Notch Resolution (*n* = 15)	Non–R‐Notch Resolution (*n* = 34)	*p* *
Age, years	73.7 ± 8.6	72.1 ± 9.1	74.3 ± 8.5	0.437
Male gender	40 (81.6%)	12 (80.0%)	28 (82.4%)	1.000
Height, cm	161.5 ± 7.7	162.4 ± 8.7	161.1 ± 7.3	0.635
Body weight, kg	58.2 [51.7, 66.9]	61.8 [54.6, 65.5]	57.6 [51.2, 67.3]	0.448
BMI, kg/m^2^	22.9 [20.1, 24.8]	23.3 [21.9, 24.4]	22.8 [19.2, 25.2]	0.632
Left ventricular ejection fraction, %	29.2 ± 3.6	29.3 ± 3.6	29.1 ± 3.7	0.870
Left ventricular end‐diastolic volume, mL	201 [167, 226]	196 [156, 270]	202 [170, 221]	0.896
Left Ventricular end‐systolic volume, mL	137 [120, 169]	133 [107, 189]	142 [122, 169]	0.745
QRS duration, ms	153.5 ± 14.9	150.1 ± 16.6	155.0 ± 14.1	0.330
Main cause of heart failure				
Ischemic cardiomyopathy	15 (30.6%)	3 (20.0%)	12 (35.3%)	0.336
Non‐ischemic cardiomyopathy	34 (69.4%)	12 (80.0%)	22 (64.7%)	
Comorbidities				
Hypertension	29 (59.2%)	10 (66.7%)	19 (55.9%)	0.542
Diabetes	12 (24.5%)	4 (26.7%)	8 (23.5%)	1.000
Dyslipidemia	32 (65.3%)	11 (73.3%)	21 (61.8%)	0.526
CKD	32 (65.3%)	7 (46.7%)	25 (73.5%)	0.104
Paroxysmal atrial fibrillation	17 (34.7%)	6 (40.0%)	11 (32.4%)	0.747
Valvular heart disease	13 (26.5%)	5 (33.3%)	8 (23.5%)	0.500
CRT‐defibrillator	44 (89.8%)	13 (86.7%)	31 (91.2%)	0.635
Medication				
ACEI/ARB/ARNI	35 (71.4%)	11 (73.3%)	24 (70.6%)	1.000
MRA	36 (73.5%)	12 (80.0%)	24 (70.6%)	0.727
Beta‐blocker	43 (87.8%)	14 (93.3%)	29 (85.3%)	0.652
Diuretic	30 (61.2%)	9 (60.0%)	21 (61.8%)	1.000
Manufacturer				0.111
Abbott	7 (14.3%)	0 (0.0%)	7 (20.6%)	
Biotronik	16 (32.7%)	7 (46.7%)	9 (26.5%)	
Medtronic	26 (53.1%)	8 (53.3%)	18 (52.9%)	

Abbreviations: ACEI, angiotensin‐converting enzyme inhibition; ARB, Angiotensin Receptor Blocker; ARNI, Angiotensin Receptor–Neprilysin Inhibitor; CKD, chronic kidney disease; CRT, cardiac resynchronization therapy.

* *p* values are for comparisons between the R‐Notch Resolution and Non–R‐Notch Resolution groups.

### 3.3 Pacing and ECG Findings

Before implantation, all patients met CLBBB criteria and showed R‐wave notching or slurring in leads V5–V6. After CRT implantation, the mean QRS duration shortened to 141.2 ± 19.2 ms. Q‐LV and LV lead position did not differ markedly between the R‐Notch Resolution and Non–R‐Notch Resolution groups (Table 2). At 1 month after implantation, the median LV‐only pacing percentage was 43.0% [0.1%–98.0%], and 26 patients (53.1%) had an LV‐only pacing percentage ≥ 50% (Table 2).

**TABLE 2:** Pacing and electrocardiographic data.

	All patients (*n* = 49)	R‐Notch Resolution (*n* = 15)	Non–R‐Notch Resolution (*n* = 34)	*p* *
QRS duration (pre‐implantation), ms	153.5 ± 14.9	150.1 ± 16.6	155.0 ± 14.1	0.33
QRS duration (post‐implantation), ms	141.2 ± 19.2	133.9 ± 17.3	144.4 ± 19.3	0.07
Q‐LV, ms	127.3 ± 34.2	129.2 ± 32.8	126.3 ± 35.6	0.80
LV lead location, *n* (%)				
Anterior	2 (4.1%)	0 (0%)	2 (5.9%)	1.00
Lateral	38 (77.6%)	13 (86.7%)	25 (73.5%)	0.46
Posterior	9 (18.4%)	2 (13.3%)	7 (20.6%)	0.70
LV lead level, *n* (%)				
Basal	9 (18.4%)	3 (20.0%)	6 (17.6%)	1.00
Mid	36 (73.5%)	12 (80.0%)	24 (70.6%)	0.73
Apical	4 (8.2%)	0 (0%)	4 (11.8%)	0.30
CLBBB (pre‐implantation), *n*	49 (100.0%)	15 (100.0%)	34 (100.0%)	
R‐wave notching or slurring in leads V5 and V6 (pre‐implantation), *n*	49 (100.0%)	15 (100.0%)	34 (100.0%)	
Resolution of R‐wave notching or slurring in leads V5 and V6 (post‐implantation), *n*	23 (46.9%)	15 (100.0%)	8 (23.5%)	—
Left ventricular only pacing at 1‐month follow‐up, %	43.0 [0.1, 98.0]	96.4 [73.0, 98.5]	1.9 [0.0, 65.1]	—
Left ventricular only pacing ≥ 50% at 1‐month follow‐up, *n*	26 (53.1%)	15 (100.0%)	11 (32.4%)	—

*Note:* Variables used for group classification are presented descriptively; therefore, no statistical testing was performed for these variables.

Abbreviation: CLBBB, complete left bundle branch block.

* *p* values are for comparisons between the R‐Notch Resolution and Non–R‐Notch Resolution groups.

### 3.4 Time Course of LVESV and LVEF

**FIGURE 3:**
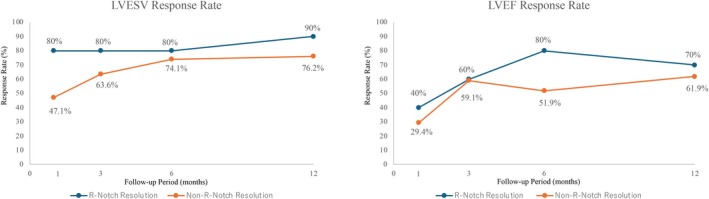
Time course of LVESV and LVEF in the R‐Notch Resolution and Non–R‐Notch Resolution groups. Left panel: Proportion of patients meeting the LVESV reverse‐remodeling criterion (≥ 15% reduction in left ventricular end‐systolic volume from baseline) at 1, 3, 6, and 12 months after CRT implantation in the R‐Notch Resolution group (*n* = 15) and the Non–R‐Notch Resolution group (*n* = 34). Right panel: Proportion of patients meeting the LVEF response criterion (≥ 5% absolute increase in left ventricular ejection fraction from baseline) at the same follow‐up time points in the two groups.

**FIGURE 4:**
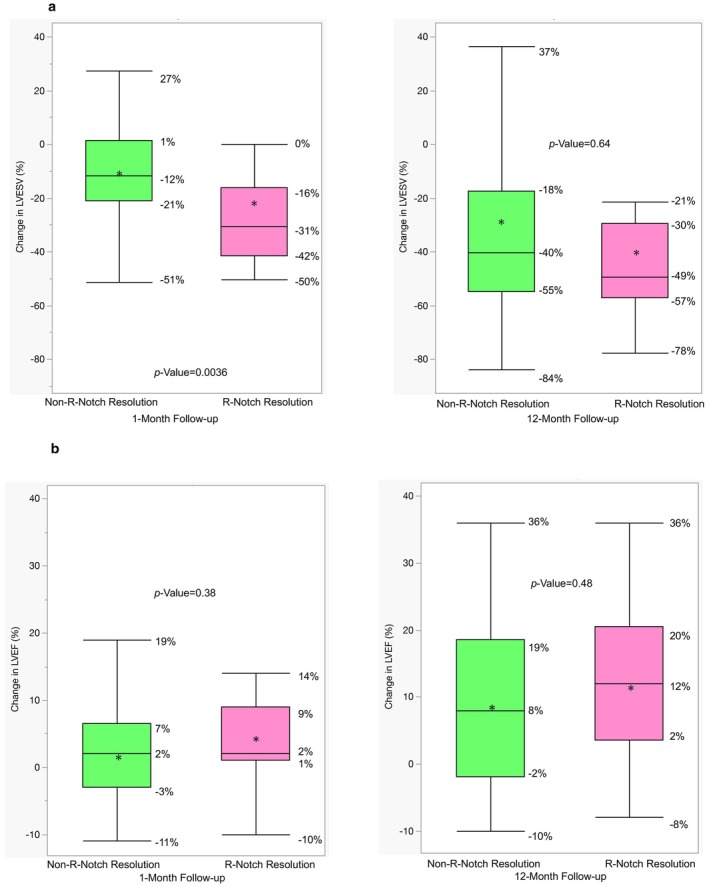
(a) Time course of relative change in LV end‐systolic volume (LVESV) according to R‐Notch Resolution and Non–R‐Notch Resolution groups. Relative change in LVESV from baseline at 1 and 12 months after CRT implantation in the R‐Notch Resolution and Non–R‐Notch Resolution groups. Negative values indicate LVESV reduction (reverse remodeling). At 1 month, the R‐Notch Resolution group showed a greater LVESV reduction than the Non–R‐Notch Resolution group (−31% vs. −12%; *p* = 0.0036), whereas by 12 months the between‐group difference was attenuated (−49% vs. −40%; *p* = 0.64). (b) Time course of relative change in left ventricular ejection fraction (LVEF) according to R‐Notch Resolution and Non–R‐Notch Resolution groups. Relative change in LVEF from baseline at 1 and 12 months after CRT implantation in the R‐Notch Resolution and Non–R‐Notch Resolution groups. Mean relative changes were 36% versus 19% at 1 month and 36% versus 20% at 12 months in the R‐Notch Resolution and Non–R‐Notch Resolution groups, respectively. Between‐group differences were not statistically significant at either time point (*p* = 0.38 and *p* = 0.48).

We compared the temporal changes in LVESV and LVEF between the R‐Notch Resolution and Non–R‐Notch Resolution groups. The proportion of patients achieving LVESV reverse remodeling at 1 month (≥ 15% reduction from baseline) was higher in the R‐Notch Resolution group than in the Non–R‐Notch Resolution group (80% vs. 47.1%) (Figure 3). With respect to the relative change in LVESV, the R‐Notch Resolution group showed a marked reduction of −31% at 1 month, compared with −12% in the Non–R‐Notch Resolution group. At 12 months, both groups exhibited substantial LVESV reduction (−49% in the R‐Notch Resolution group and −40% in the Non–R‐Notch Resolution group), and the between‐group difference was less pronounced (Figure 4a). LVEF improved over time in both groups, with a numerically greater improvement in the R‐Notch Resolution group, although the between‐group differences did not reach statistical significance at any time point (Figure 4b).

### 3.5 Factors Associated With 1‐Month LVESV Reverse Remodeling

In univariable logistic regression analysis with 1‐month LVESV reverse remodeling as the dependent variable, R‐Notch Resolution group status and QRS shortening were associated with 1‐month LVESV reverse remodeling, whereas pre‐CRT QRS duration and post‐CRT QRS duration were not significantly associated with this endpoint (Table 3). QRS shortening was defined as pre‐CRT QRS duration minus post‐CRT QRS duration.

**TABLE 3:** Univariable analysis of factors associated with 1‐month LVESV reverse remodeling.

Variables	Univariable	
	OR (95% CI)	*p*
R‐Notch Resolution group	1.7 (1.1 to 2.63)	0.032
Female	0.96 (0.50 to 1.84)	0.92
Non‐ischemic cardiomyopathy	1.2 (0.67 to 2.16)	0.52
Pre‐CRT QRS duration	1.03 (0.99 to 1.07)	0.14
Post‐CRT QRS duration	0.98 (0.95 to 1.01)	0.23
ΔQRS	1.03 (1.00 to 1.06)	0.04
Age	1.01 (0.94 to 1.08)	0.73
Height	0.98 (0.9 to 1.06)	0.63
LV‐only pacing percentage, per 1% increase	1.01 (0.99 to 1.03)	0.08

Abbreviations: CI, confidence interval; OR, odds ratio.

In the main multivariable analysis, R‐Notch Resolution group status remained independently associated with 1‐month LVESV reverse remodeling (Table 4). Because QRS shortening and notch/slur resolution are mechanistically related ECG markers of electrical resynchronization, and because of the limited number of events, these variables were not entered simultaneously into the same multivariable model. Therefore, we performed an additional exploratory multivariable analysis in which QRS shortening was entered into the model instead of R‐Notch Resolution group status. In this alternative exploratory model, QRS shortening did not remain significantly associated with 1‐month LVESV reverse remodeling after adjustment for covariates (Table S2).

**TABLE 4:** Multivariable analysis of factors associated with 1‐month LVESV reverse remodeling.

Variables	Multivariable	
	OR (95% CI)	*p*
R‐Notch Resolution group	6.7 (1.52 to 40.9)	0.011
Female	0.43 (0.04 to 4.48)	0.48
Non‐ischemic cardiomyopathy	1.17 (0.22 to 5.65)	0.85
Pre‐CRT QRS duration	1.04 (0.99 to 1.09)	0.16
Age	0.97 (0.88 to 1.01)	0.56
Height	0.95 (0.83 to 1.08)	0.45

Abbreviations: CI, confidence interval; OR, odds ratio.

## 4 Discussion

### 4.1 Main Findings of the Present Study

In this study of patients with heart failure and CLBBB undergoing CRT, we found that patients who (1) showed resolution of R‐wave notching/slurring in leads V5–V6 under LV‐only pacing at implantation and (2) had an LV‐only pacing rate ≥ 50% at 1 month (R‐Notch Resolution group) exhibited a significantly greater reduction in LVESV as early as 1 month after CRT implantation compared with the Non–R‐Notch Resolution group. Membership in the R‐Notch Resolution group was independently associated with LVESV reverse remodeling at 1 month. Traditionally, CRT response has mainly been evaluated using LVRR or improvement in LVEF at 6–12 months [4, 8, 18, 19]. In contrast, the present study suggests that a simple ECG finding—resolution of QRS notching/slurring during LV‐only pacing—when combined with an LV‐only pacing percentage ≥ 50% at 1 month, may serve as a marker of early structural improvement after CRT implantation, representing a novel aspect of our work.

### 4.2 Significance of QRS Notch/Slur Resolution Under LV‐Only Pacing

In patients with LBBB, R‐wave notching or slurring in the lateral leads is a characteristic morphological feature and is incorporated into criteria for identifying “true complete LBBB” morphology. In particular, patients who fulfill the so‐called true complete LBBB morphology tend to show a favorable response to CRT, suggesting that QRS morphology, including lateral notching and slurring, is an important determinant of the potential for electrical resynchronization [11, 12, 13, 14]. In addition, several studies have shown that CRT‐induced QRS narrowing is associated with acute hemodynamic improvement and long‐term left ventricular reverse remodeling (LVRR) [9, 10, 23]. In particular, Lecoq et al. identified acute QRS shortening as one of the strongest ECG predictors of a favorable CRT response [24]. In the present study, the R‐Notch Resolution group consisted of patients in whom V5–V6 notching/slurring resolved under LV‐only pacing and who also achieved a sufficiently high LV‐only pacing rate. This pattern is likely to reflect not merely the additive effects of two separate markers, but rather an integrated electrophysiological state characterized by effective LV‐only fusion pacing and improved intraventricular synchrony. In this state, preserved intrinsic atrioventricular conduction allows LV‐paced activation to fuse with native right bundle branch conduction, while resolution of QRS notching/slurring suggests normalization of delayed lateral left ventricular activation. Therefore, the coexistence of a high LV‐only pacing rate and notch/slur resolution may identify patients in whom optimal electrical resynchronization has been achieved. The marked reduction in LVESV observed as early as 1 month in the R‐Notch Resolution group supports the notion that resolution of QRS notching/slurring represents not only simple QRS narrowing but also more complete normalization of ventricular activation. Taken together, these findings suggest that resolution of QRS notching/slurring under LV‐only pacing, particularly when accompanied by a high LV‐only pacing rate, may serve as a useful electrophysiological marker not only for identifying responders, but specifically for identifying patients in whom the beneficial effects of CRT are likely to manifest early.

### 4.3 Clinical Implications of Early Reverse Remodeling

LVRR induced by CRT typically develops over several months to a year, and the extent of this remodeling is known to be closely associated with long‐term prognosis [4, 8, 18, 19]. Conversely, it has also been reported that acute QRS shortening immediately after CRT initiation is associated with subsequent long‐term mortality, and Jastrzębski et al. demonstrated in patients with LBBB that CRT‐induced QRS narrowing was associated with more favorable long‐term outcomes [25]. Taken together, these findings suggest that early electrical resynchronization may serve as an “early marker” of subsequent structural improvement and clinical outcomes. In the present study, LVESV reverse remodeling was frequently observed as early as 1 month after CRT implantation in patients who exhibited resolution of notching/slurring and a high LV‐only pacing rate. These results indicate that the quality of electrical resynchronization can translate into structural improvement within a relatively short period of time, supporting the value of assessing notch resolution during early follow‐up. Although the between‐group separation in reverse remodeling was most apparent at 1 month and attenuated during longer follow‐up, this does not necessarily negate the clinical utility of an early marker. In patients with advanced heart failure, even modest early improvement may facilitate stabilization during the vulnerable post‐implant period and help guide timely reassessment and optimization when symptoms remain refractory. The reduced separation at later time points may reflect temporal variability in electrical resynchronization and fusion pacing, including reappearance of notching/slurring in some patients, together with the absence of a protocolized re‐optimization strategy in this retrospective cohort. Notably, established predictors in larger CRT cohorts (e.g., female sex and non‐ischemic etiology) were not statistically significant in our analysis, likely reflecting the small sample size and the focus on an early (1‐month) endpoint; the corresponding estimates showed wide confidence intervals (Table 3). Clinically, in patients who show notch resolution and LVESV improvement at 1 month, it may be reasonable to maintain the current programming and continue observation, whereas in those with persistent notching and limited LVESV improvement, early reconsideration of device programming, optimization of medical therapy, and evaluation of concomitant conditions such as ischemia or tachyarrhythmias should be considered.

### 4.4 LV‐Only Pacing and Implications for Programming Optimization

LV‐only pacing is a resynchronization strategy that achieves fusion between intrinsic right ventricular conduction and left ventricular pacing, and has been shown to be non‐inferior to conventional BVP with respect to improvements in cardiac function and NYHA class [15, 16, 17, 26]. Studies of LV‐only fusion pacing have reported that higher LV‐only pacing percentages are associated with greater CRT benefit [15, 22]. In this context, the R‐Notch Resolution group in our study—defined by both resolution of notching/slurring and an LV‐only pacing rate ≥ 50%—can be regarded as a subset in which a high degree of fusion pacing was achieved, and our findings suggest that in such patients the beneficial effects of CRT may emerge earlier than traditionally expected. In clinical practice, LV‐only fusion pacing is influenced by both device programming and physiological conditions, including intrinsic atrioventricular conduction and heart rate, and recent data suggest that fused AV‐delay optimization may improve the acute hemodynamic response during CRT [27]. Vendor‐specific algorithms such as AdaptiveCRT and AutoAdapt generally determine the pacing mode based on intrinsic AV conduction and its stability. LV‐only pacing is favored when intrinsic AV conduction is sufficiently preserved and stable within the algorithm‐defined criteria, allowing fusion between native right ventricular activation and LV‐paced activation. Conversely, when intrinsic AV conduction is prolonged, unstable, or unsuitable for maintaining effective fusion, biventricular pacing tends to be selected or become more dominant. In contrast, SyncAV was used for AV delay optimization to achieve QRS narrowing and does not inherently provide LV‐only pacing. Therefore, the maintenance of a high LV‐only pacing percentage depends not only on the selected algorithm or programmed settings, but also on the stability of intrinsic conduction over time. The persistence of R‐wave notching/slurring during LV‐only pacing may reflect incomplete electrical resynchronization. LV‐only pacing combines intrinsic right ventricular activation through the right bundle branch with LV‐paced activation; therefore, notch/slur resolution may indicate not only improved intraventricular LV activation but also more favorable global ventricular synchrony. Consistent with this concept, greater QRS shortening has been associated with an improved echocardiographic response after resynchronization pacing [28]. Conversely, persistent notching/slurring may suggest insufficient fusion due to suboptimal AV timing, residual LV conduction disturbance, myocardial scar, or an unfavorable LV lead position. This may partly explain the early LVESV reduction observed in the R‐Notch Resolution group, although this mechanism remains speculative because ventricular activation sequence and mechanical dyssynchrony were not directly assessed. In contrast, BiV pacing, particularly when combined with algorithms incorporating intrinsic conduction, may produce a more complex activation pattern involving intrinsic conduction, RV pacing, and LV pacing. Therefore, even apparently optimal BiV pacing may not necessarily result in complete normalization of lateral LV activation or resolution of QRS notching/slurring. In patients programmed with BiV pacing, R‐wave notch/slur resolution was not achieved under the programmed BiV pacing condition; therefore, these patients were classified into the Non–R‐Notch Resolution group. These findings should not be interpreted as showing the universal superiority of LV‐only pacing over BiV fusion pacing strategies, but rather as suggesting that LV‐only fusion pacing with notch/slur resolution may identify patients with early effective electrical resynchronization. This study was a retrospective analysis based on clinical practice, in which vendor‐specific algorithms and AV/VV delay settings were chosen at the discretion of the treating physicians; programming was not prospectively directed toward achieving notch resolution. Nonetheless, the observed association between the combination of notch resolution and a high LV‐only pacing rate and early LV reverse remodeling provides useful insight for future programming strategies. It should also be emphasized that our findings do not support the routine preferential use of LV‐only pacing regardless of device‐based recommendations or patient‐specific conduction status. In this study, LV‐only pacing was used within routine clinical practice with vendor‐specific algorithms designed to incorporate intrinsic conduction. Accordingly, assessment of R‐wave notch/slur resolution may be most applicable when LV‐only pacing is supported by the device algorithm and stable fusion with intrinsic right ventricular conduction is expected. The present findings should not be extrapolated to patients in whom LV‐only fusion pacing is inappropriate or difficult to maintain, such as those with high‐grade atrioventricular block or absent intrinsic conduction. In particular, algorithms such as AutoAdapt (Biotronik) and SyncAV plus (Abbott), which allow fine‐tuning of the timing of LV pacing relative to intrinsic conduction, may be useful for optimizing LV‐only fusion pacing in clinical practice [20, 21]. In addition, some patients in our cohort exhibited reappearance of notching/slurring after having initially shown notch resolution. This suggests that the degree of electrical resynchronization is not fixed, but may fluctuate over time in response to changes in heart rate, AV conduction, medical therapy, and device settings. Stable LV‐only fusion pacing may also be influenced by physiological factors such as heart rate and intrinsic atrioventricular conduction, both of which can be modified by medical therapy. Although pharmacological optimization may theoretically help maintain an appropriate fusion window, medication adjustment was not performed according to a predefined protocol in this retrospective study. Therefore, the impact of medical therapy on the stability of LV‐only fusion pacing could not be assessed and should be evaluated in future prospective studies. Moving forward, it will be important to design interventional protocols in which the reappearance of notching is used as a trigger for reassessing AV delay and re‐optimizing LV‐only fusion pacing, and to prospectively evaluate whether such strategies improve outcomes.

## 5 Limitations

This study has several limitations. First, it was a single‐center retrospective analysis with a relatively small sample size of 49 patients; therefore, selection bias and residual confounding cannot be completely excluded. However, we screened consecutive CRT recipients using predefined criteria, and baseline characteristics were comparable between included and excluded patients (Table S1). In addition, the cohort was relatively small and predominantly male, which likely limited the statistical power to detect associations between established favorable factors, such as female sex and nonischemic etiology [29], and early left ventricular reverse remodeling. Consistent with this, the regression estimates for these variables showed wide confidence intervals (Table 3), indicating limited precision. Second, the use of LV‐only pacing algorithms and the detailed programming of AV/VV delay were largely left to the discretion of the treating physicians, and the true degree of fusion could not be uniformly assessed. Moreover, LV‐only pacing percentage was mainly evaluated at the 1‐month time point, and temporal changes thereafter were not fully analyzed.

In addition, the cutoff value of LV‐only pacing burden was not based on an established standard definition; the threshold of ≥ 50% was selected pragmatically to indicate predominant LV‐only pacing during follow‐up and was informed by the published post hoc analysis of the Adaptive CRT trial by Birnie et al. [30] Third, the presence of QRS notching/slurring was assessed visually and thus cannot be regarded as entirely objective. In this study, ECGs were evaluated independently by multiple readers and final decisions were made by consensus; however, future studies using vectorcardiography or automated ECG analysis would be desirable to achieve more objective assessment. Finally, the primary endpoint was early change in LVESV, and hard clinical outcomes such as heart failure rehospitalization and all‐cause mortality were not comprehensively evaluated; prospective studies are needed to clarify the relationship between notch resolution and long‐term prognosis.

## 6 Conclusion

In patients with heart failure and CLBBB undergoing CRT, those in whom R‐wave notching/slurring in leads V5–V6 resolved under LV‐only pacing and who achieved an LV‐only pacing rate ≥ 50% at 1 month were more likely to exhibit left ventricular end‐systolic volume reverse remodeling as early as 1 month after implantation.

QRS notch/slur resolution during LV‐only fusion pacing is a simple parameter that can be assessed using a standard 12‐lead ECG and, when accompanied by an LV‐only pacing percentage ≥ 50%, may serve as a useful marker associated with early CRT response and may help guide post‐implant optimization of device programming. Future prospective studies are warranted to confirm the reproducibility of these findings, to clarify their relationship with long‐term outcomes, and to evaluate the efficacy of programming strategies that aim to achieve and maintain notch resolution.

## Ethics Statement

The study protocol was approved by the Institutional Review Board of Sapporo Cardiovascular Clinic (approval number: C25‐1‐02).

## Consent

Because of the retrospective nature of the study, the requirement for individual written informed consent was waived, and patients were provided with the opportunity to opt out through the institutional website.

## Conflicts of Interest

Dr. Kondo received lecture fees from Daiichi‐Sankyo, Medtronic, Abbott Medical Japan, Biotronik, Boston Scientific, and Japan Lifeline, and research funds from Daiichi‐Sankyo and Boston Scientific. Other authors have no conflicts of interest to declare.

## Supporting information


**Table S1:** Baseline characteristics of included and excluded patients.

**Table S2:** Alternative multivariable model including QRS shortening for predicting 1‐month LVESV reverse remodeling.

## Data Availability

The data that support the findings of this study are available on request from the corresponding author. The data are not publicly available due to privacy or ethical restrictions.
