# Supplementary material for: Association of QRS Notch Resolution During LV‐Only Fusion Pacing With Early Left Ventricular Reverse Remodeling After Cardiac Resynchronization Therapy
Source: J Arrhythm. 2026 Aug 9;42(4):e70443. doi: 10.1002/joa3.70443 (PMC13454736; doi:10.1002/joa3.70443)
Supplement: Supplementary file 1 [file JOA3-42-e70443-s001.docx]

| **Supplementary Table S1. Baseline characteristics of included and excluded patients** | | | | |
| --- | --- | --- | --- | --- |
|  | **Included** (n = 49) | **Excluded** (n = 40) | p Value |  |
| Age, years | 73.7 ± 8.6 | 71.4 ± 13.4 | 0.33 |  |
| Male gender | 40 (81.6%) | 30 (75.0%) | 0.62 |  |
| Height, cm | 161.5 ± 7.7 | 162.1 ± 8.1 | 0.74 |  |
| Body weight, kg | 58.2 [51.4, 67.2] | 58.2 [55.0, 68.5] | 0.53 |  |
| Body mass index, kg/m² | 22.9 [20.0, 25.1] | 22.5 [21.2, 24.7] | 0.79 |  |
| Left ventricular ejection fraction, % | 29.2 ± 3.6 | 31.6 ±7.4 | 0.79 |  |
| Left ventricular end-diastolic volume, ml | 201 [163, 232] | 174 [148, 218] | 0.11 |  |
| Left Ventricular end-systolic volume, ml | 137 [114, 171] | 118 [97, 157] | 0.08 |  |
| QRS duration, ms | 153.5 ± 14.9 | 156 ± 12.4 | 0.28 |  |
| Main cause of heart failure |  |  |  |  |
| Ischemic cardiomyopathy | 15 (30.6 %) | 10 (25.0%) | 0.73 |  |
| Non-ischemic cardiomyopathy | 34 (69.4 %) | 30 (75.0%) | 0.73 |  |
| Comorbidities |  |  |  |  |
| Hypertension | 29 (59.2 %) | 16 (40.0%) | 0.11 |  |
| Diabetes | 12 (24.5 %) | 16 (40.0%) | 0.18 |  |
| Dyslipidemia | 32 (65.3 %) | 24 (60.0%) | 0.77 |  |
| CKD | 32 (65.3 %) | 28 (70.0%) | 0.81 |  |
| Paroxysmal atrial fibrillation | 17 (34.7 %) | 7 (17.5%) | 0.45 |  |
| Valvular heart disease | 13 (26.5 %) | 11 (27.5%) | 1.00 |  |
| Medication |  |  |  |  |
| ACEI/ARB/ARNI | 35 (71.4%) | 20 (50.0%) | 0.06 |  |
| MRA | 37 (75.5%) | 31 (77.5%) | 1.00 |  |
| Beta-blocker | 45 (91.8%) | 34 (85.0%) | 0.34 |  |
| Diuretic | 33 (67.3%) | 28 (70.0%) | 0.97 |  |
| CKD (chronic kidney disease) ACEI (angiotensin-converting enzyme inhibition) ARB (Angiotensin Receptor Blocker) | | | | |
