# Supplementary material for: Association of QRS Notch Resolution During LV‐Only Fusion Pacing With Early Left Ventricular Reverse Remodeling After Cardiac Resynchronization Therapy
Source: J Arrhythm. 2026 Aug 9;42(4):e70443. doi: 10.1002/joa3.70443 (PMC13454736; doi:10.1002/joa3.70443)
Supplement: Supplementary file 2 [file JOA3-42-e70443-s002.docx]

| **Table S2. Alternative Multivariable Model Including QRS Shortening for Predicting 1-Month LVESV Reverse Remodeling** | | |
| --- | --- | --- |
| Variables | Multivariable | |
|  | OR (95% CI) | *p*-Value |
| ΔQRS | 1.02 (0.99 to 1.07) | 0.13 |
| Female | 0.41 (0.04 to 4.12) | 0.44 |
| Non-ischemic cardiomyopathy | 1.38 (0.30 to 6.08) | 0.67 |
| Pre-CRT QRS duration | 1.00 (0.95 to 1.05) | 0.93 |
| Age | 0.97 (0.89 to 1.07) | 0.59 |
| Height | 0.97 (0.86 to 1.10) | 0.67 |
| CI, confidence interval; OR, odds ratio | | |
